# Supplementary figures and images for: Forty new genomes shed light on sexual reproduction and the origin of tetraploidy in Microsporidia
Source: PLoS Biol. 2025 Oct 21;23(10):e3003446. doi: 10.1371/journal.pbio.3003446 (PMC12558613; doi:10.1371/journal.pbio.3003446)

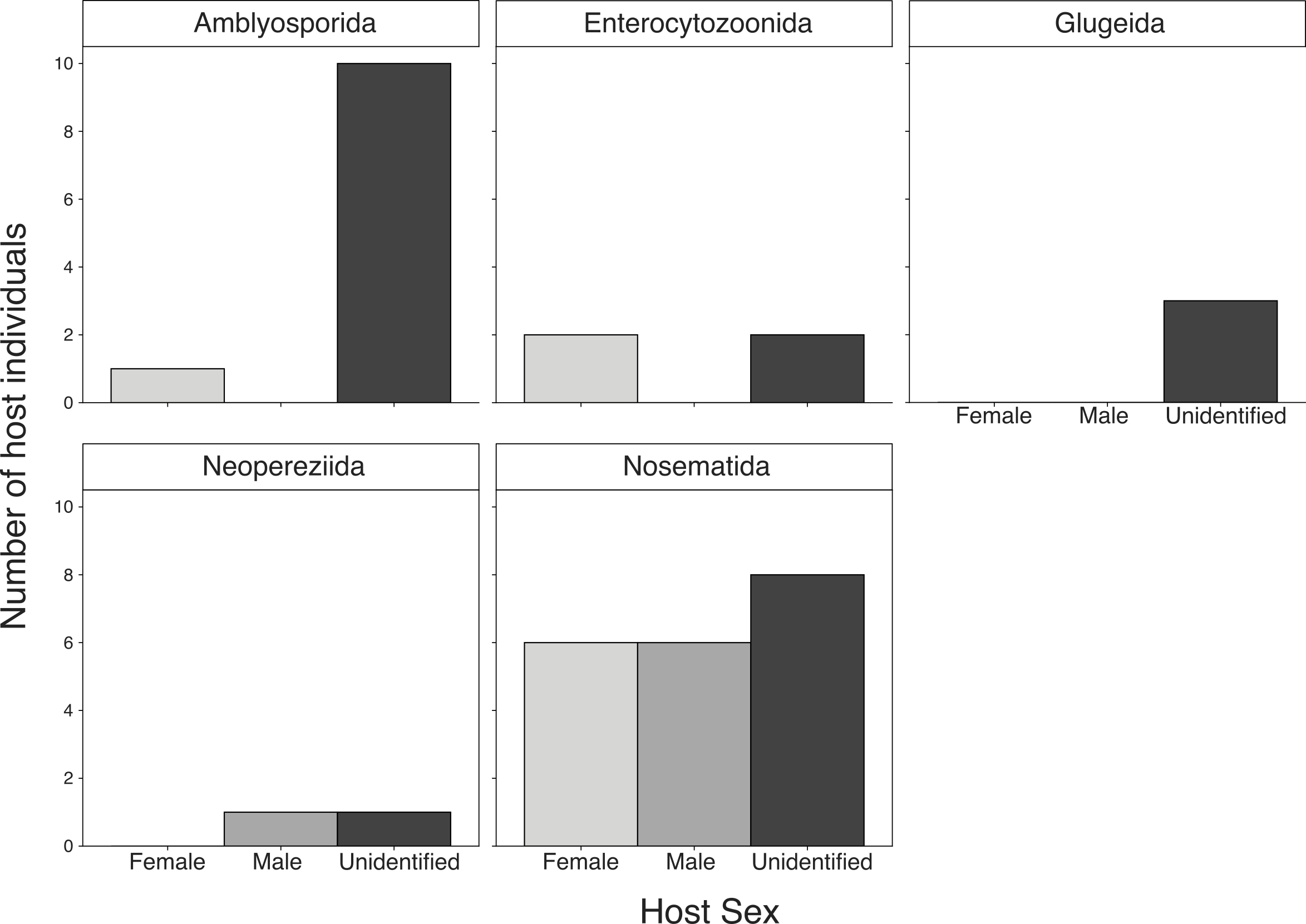

Supplement: S1 Fig — The sex of our genomes’ hosts was unknown in most cases (24 species). In the remaining cases, nine were identified as female and seven as male. A relatively equal proportion of female and male hosts are infected with Nosematida (Fig 1), but we could not assess skews in host sex ratios for other microsporidian groups due to missing data on sex (for Amblyosporida-infected hosts), or a small sample size (for Neopereziida-infected hosts). The data underlying this figure can be found in S1 Table. The figure was generated using Matplotlib [92], and manually annotated using InkScape (version 1.2.2). (PNG) [file pbio.3003446.s011.png]

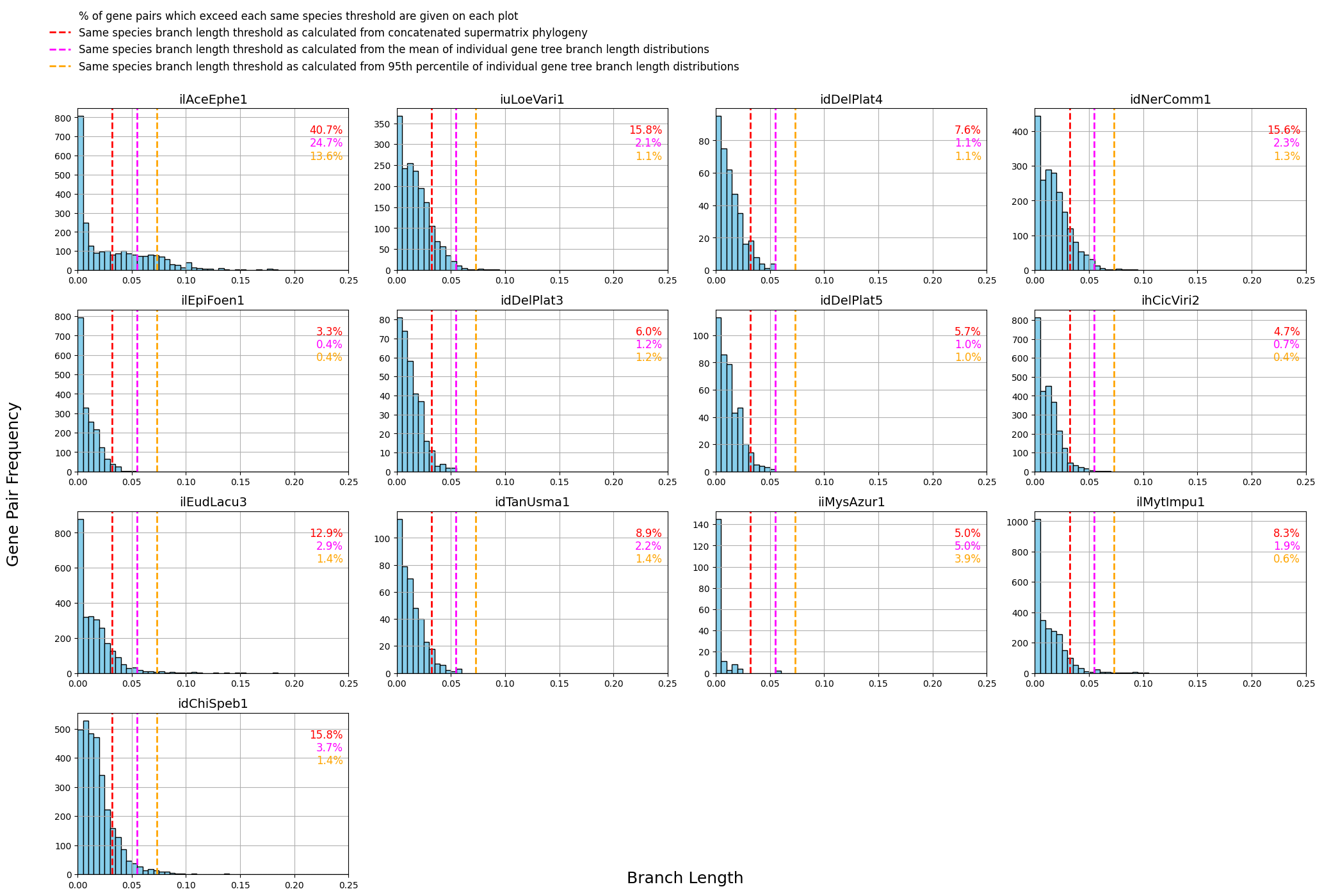

Supplement: S4 Fig — The approach we presented in the main text relies on branch lengths derived from the whole-genome phylogeny in Fig 2 (i.e., a concatenated supermatrix of genes). We re-estimated same-species branch length thresholds for each gene. For each gene, we used the distribution of branch lengths between genomes known to belong to the same species, and measured each distribution’s mean and 95th percentile. The upper threshold was then set by retrieving the highest observed 95th percentile (orange dashed line) and the highest observed mean (magenta dashed line). While the percentage of genes exceeding each threshold varies for each genome, they are relatively consistent, and lead to the same OTU assignment and the same conclusions when investigating tetraploid species. ilAceEphe1.µ still stands out as possessing more genes which exceed the same-species threshold (no matter what threshold was used) than other genomes. The figure was generated using Matplotlib [92], and manually annotated using InkScape (version 1.2.2). (PNG) [file pbio.3003446.s014.png]

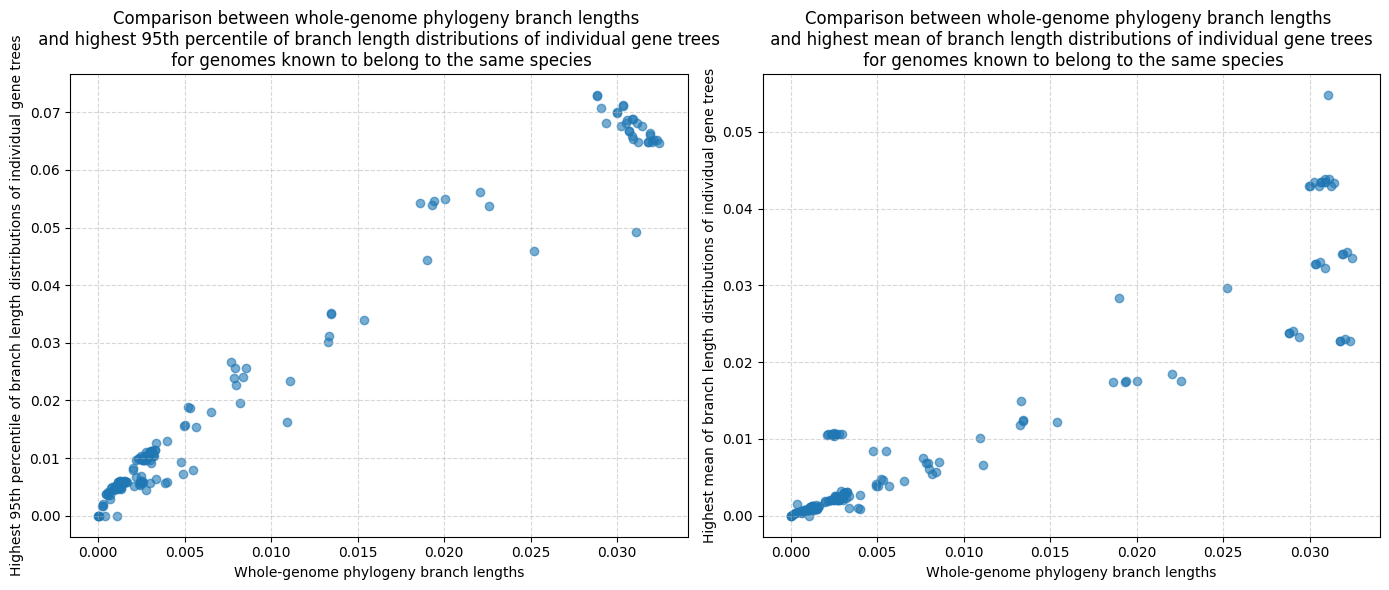

Supplement: S5 Fig — We compared our two gene-based metrics (highest 95th percentile and highest mean of branch length distributions of individual gene trees for genomes known to belong to the same species) to the whole-genome-based metric (highest branch length observed between any two same species genomes). We found the relationship between them to be consistent and linear, in line with the fact that they lead to the same conclusions. The figure was generated using Matplotlib [92], and manually annotated using InkScape (version 1.2.2). (PNG) [file pbio.3003446.s015.png]

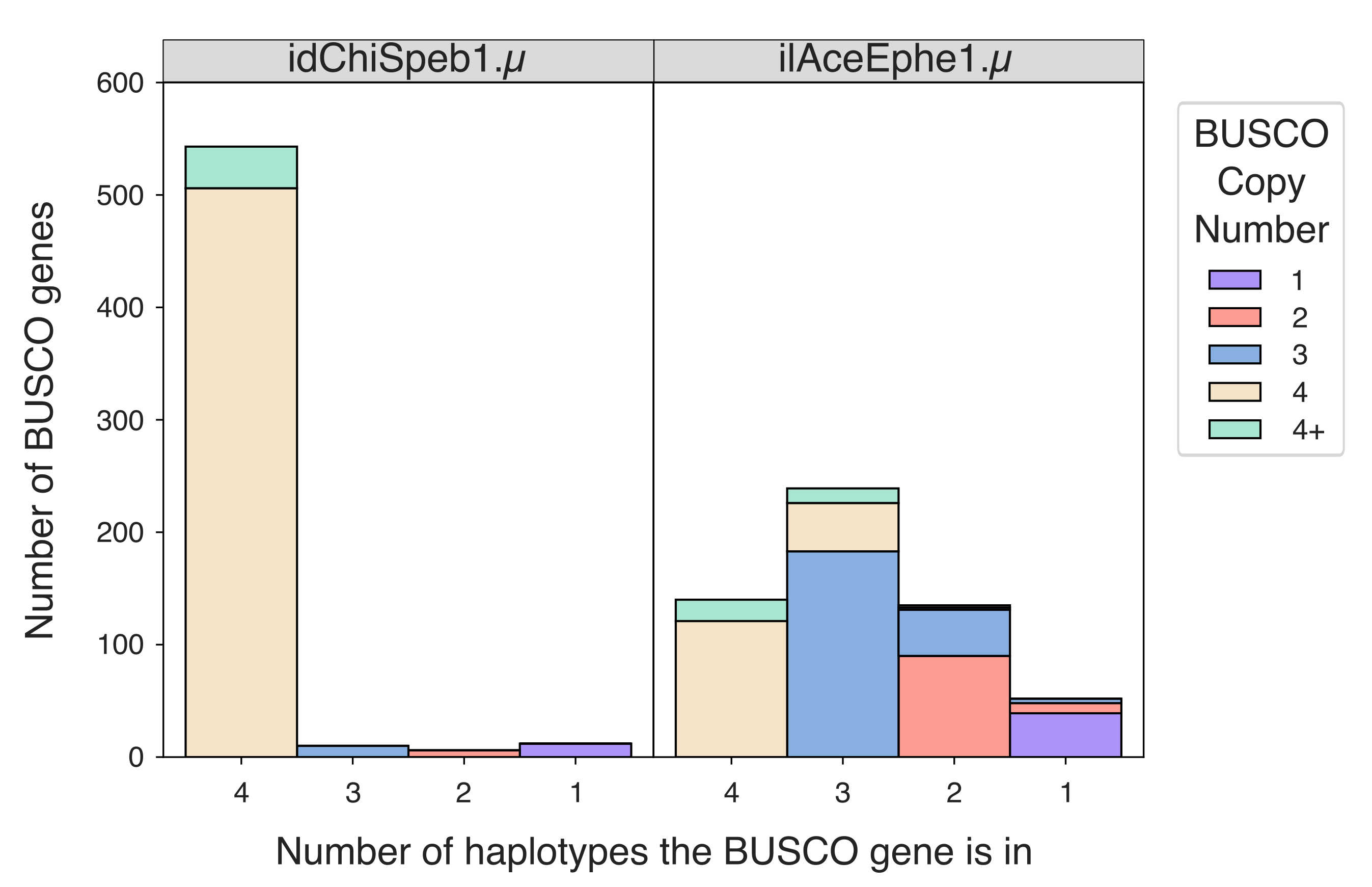

Supplement: S6 Fig — The number of BUSCO genes found in X haplotypes, along with their total copy number. idChiSpeb1.µ is an even tetraploid, so nearly all its BUSCO genes are in 4 copies, distributed across 4 haplotypes. On the other hand, ilAceEphe1.µ is an uneven tetraploid. The majority of its BUSCO genes are in less than 4 copies, and they are not evenly distributed across its haplotypes. For instance, some BUSCO genes occur in 3 copies present only in a single haplotype. The figure was generated using gerbil (Github: https://github.com/Amjad-Khalaf/gerbil), and manually annotated using InkScape (version 1.2.2). (PNG) [file pbio.3003446.s016.png]

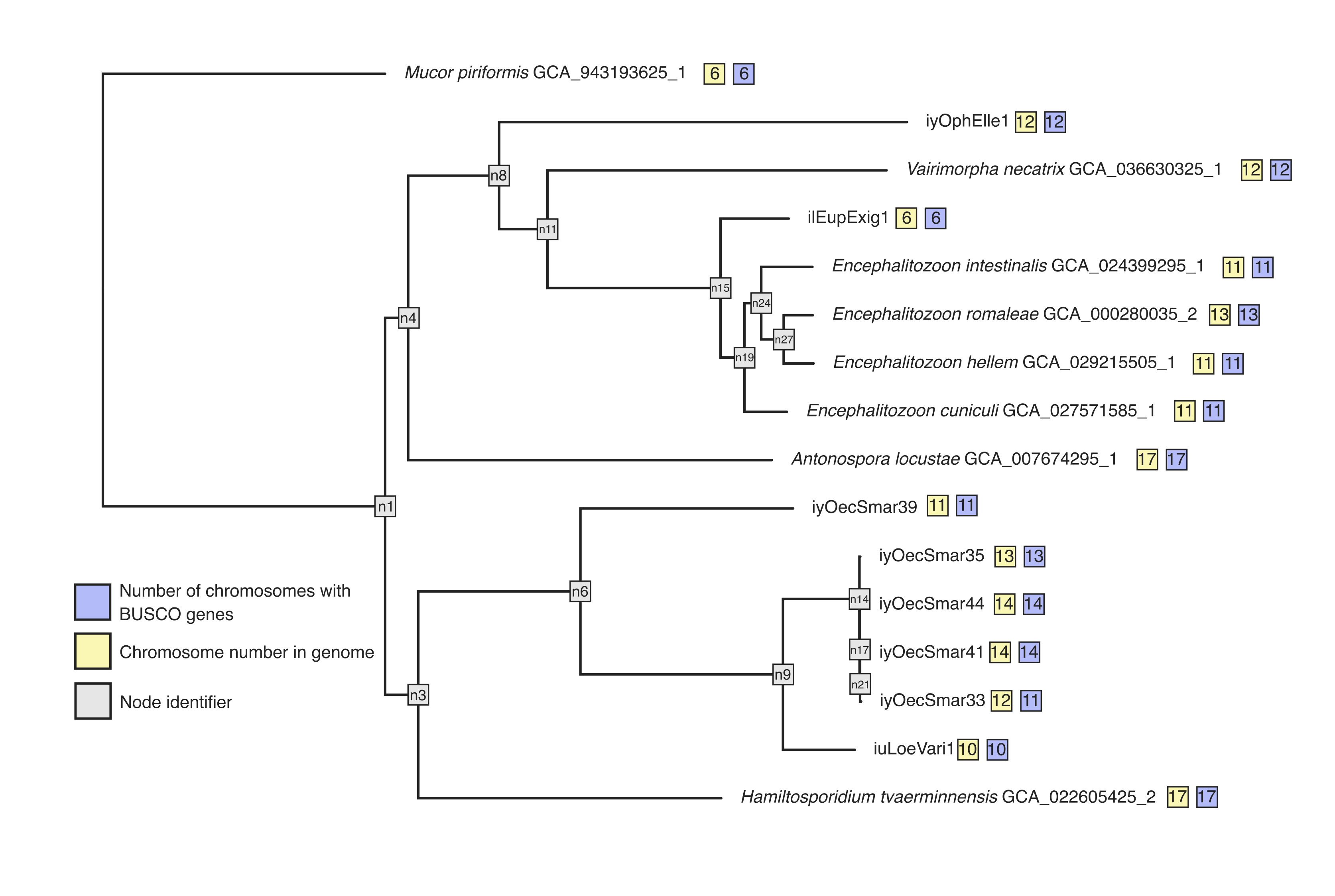

Supplement: S7 Fig — Each node is labeled with its Syngraph name in a gray box. Yellow boxes indicate the number of chromosomes each genome possesses, and blue boxes indicate the number of chromosomes which possess BUSCO gene markers. The figure was generated using ToyTree [73], and manually annotated using InkScape (version 1.2.2). (PNG) [file pbio.3003446.s017.png]

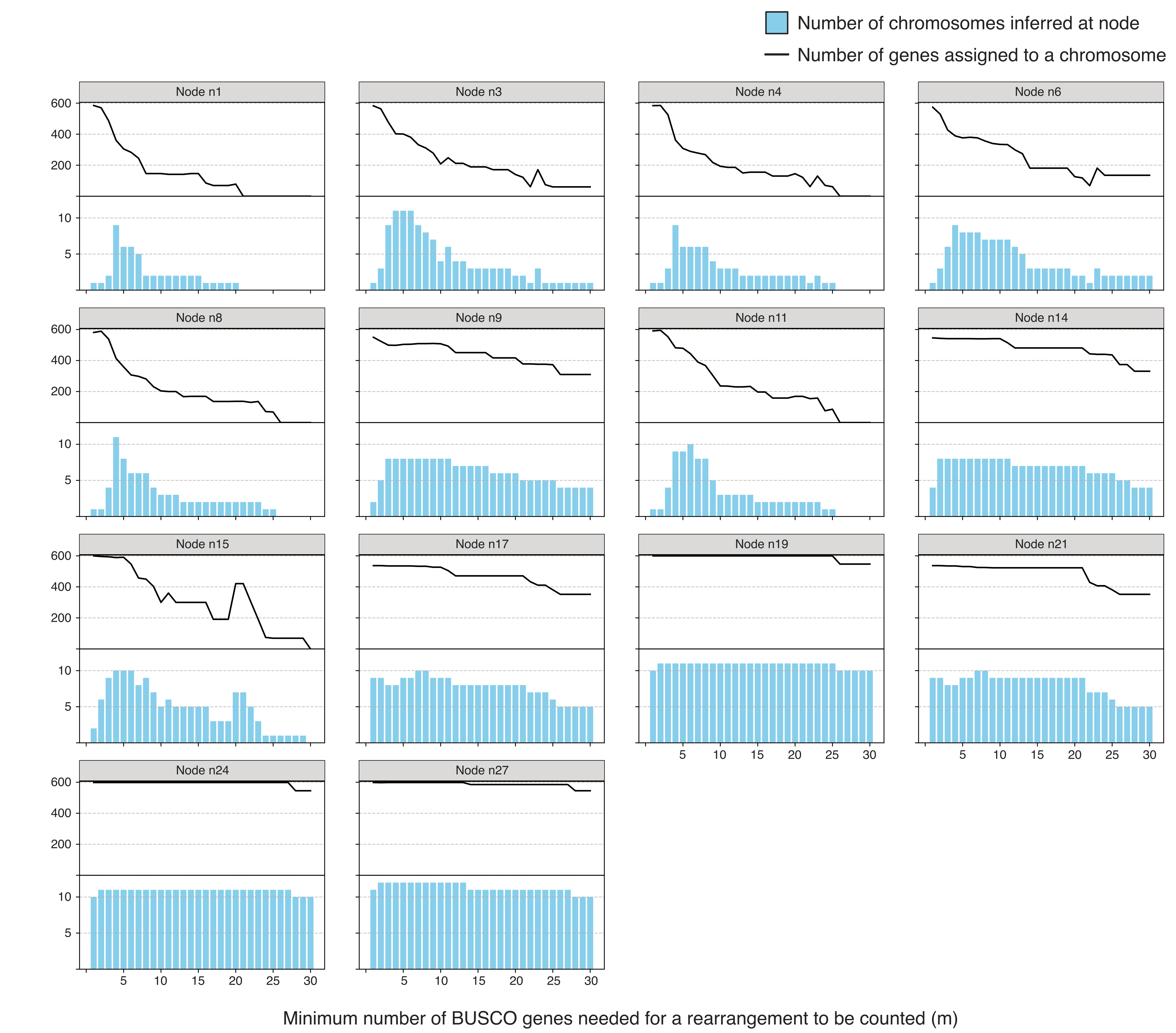

Supplement: S8 Fig — The number of chromosomes inferred for each node, and the total number of BUSCO genes assigned to a chromosome for each “m.” “m” is the parameter in Syngraph to determine the minimum number of genes needed to travel together for the event to be counted as a rearrangement. For example, if m = 3, only rearrangements involving 3 or more genes will be counted. Deep nodes are highly variable and their karyotype (and thus the number of rearrangements that have occurred along each branch) cannot be estimated reliably. See S7 Fig for node labels on the phylogeny. The figure was generated using Matplotlib [92], and manually annotated using InkScape (version 1.2.2). (PNG) [file pbio.3003446.s018.png]

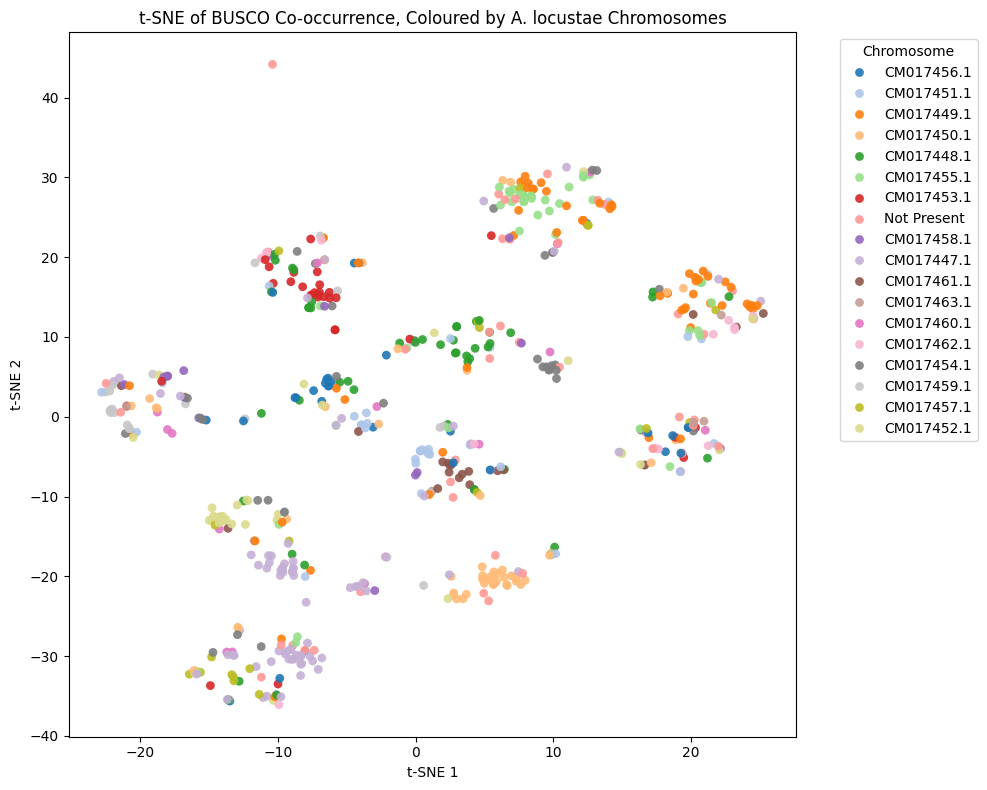

Supplement: S9 Fig — Each point represents a BUSCO gene, positioned based on its co-occurrence profile across the chromosome-level microsporidian genomes. Distances between points reflect similarities in co-occurrence. Points are coloured by their assigned chromosome in Anotonspora locustae. This disorganized pattern illustrates that the rate of rearrangement is too high for a reliable complete reconstruction of putative ancestral linkage groups. The large-scale patterns are influenced by more densely sampled taxa, see S10 Fig. The data underlying this figure can be found in File Collection 5 at https://doi.org/10.5281/zenodo.17251512. The figure was generated using Scikit-learn [142,143] and Matplotlib [92], and manually annotated using InkScape (version 1.2.2). (PNG) [file pbio.3003446.s019.png]

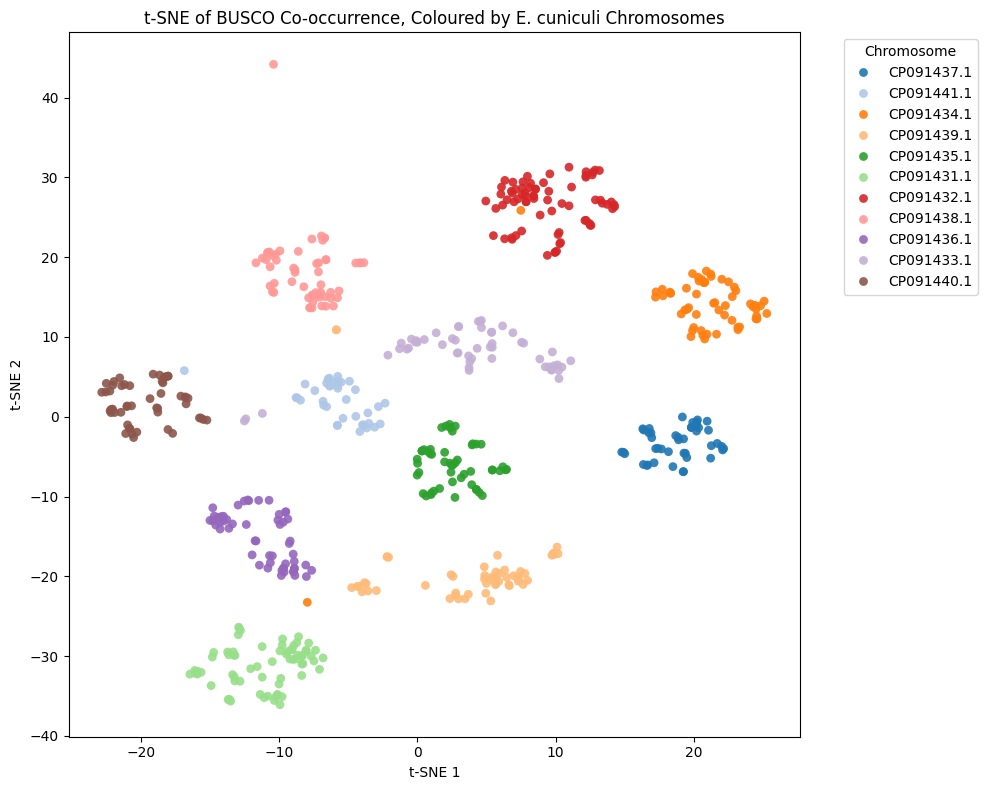

Supplement: S10 Fig — Each point represents a BUSCO gene, positioned based on its co-occurrence profile across the chromosome-level microsporidian genomes. Distances between points reflect similarities in co-occurrence. Points are coloured by their assigned chromosome in Encephalitozoon cuniculi. This disorganized pattern illustrates that the rate of rearrangement is too high for a reliable complete reconstruction of putative ancestral linkage groups. The large-scale patterns are influenced by more densely sampled taxa, such as Encephalitozoon cuniculi. The data underlying this figure can be found in File Collection 5 at https://doi.org/10.5281/zenodo.17251512. The figure was generated using Scikit-learn [142,143] and Matplotlib [92], and manually annotated using InkScape (version 1.2.2). (PNG) [file pbio.3003446.s020.png]

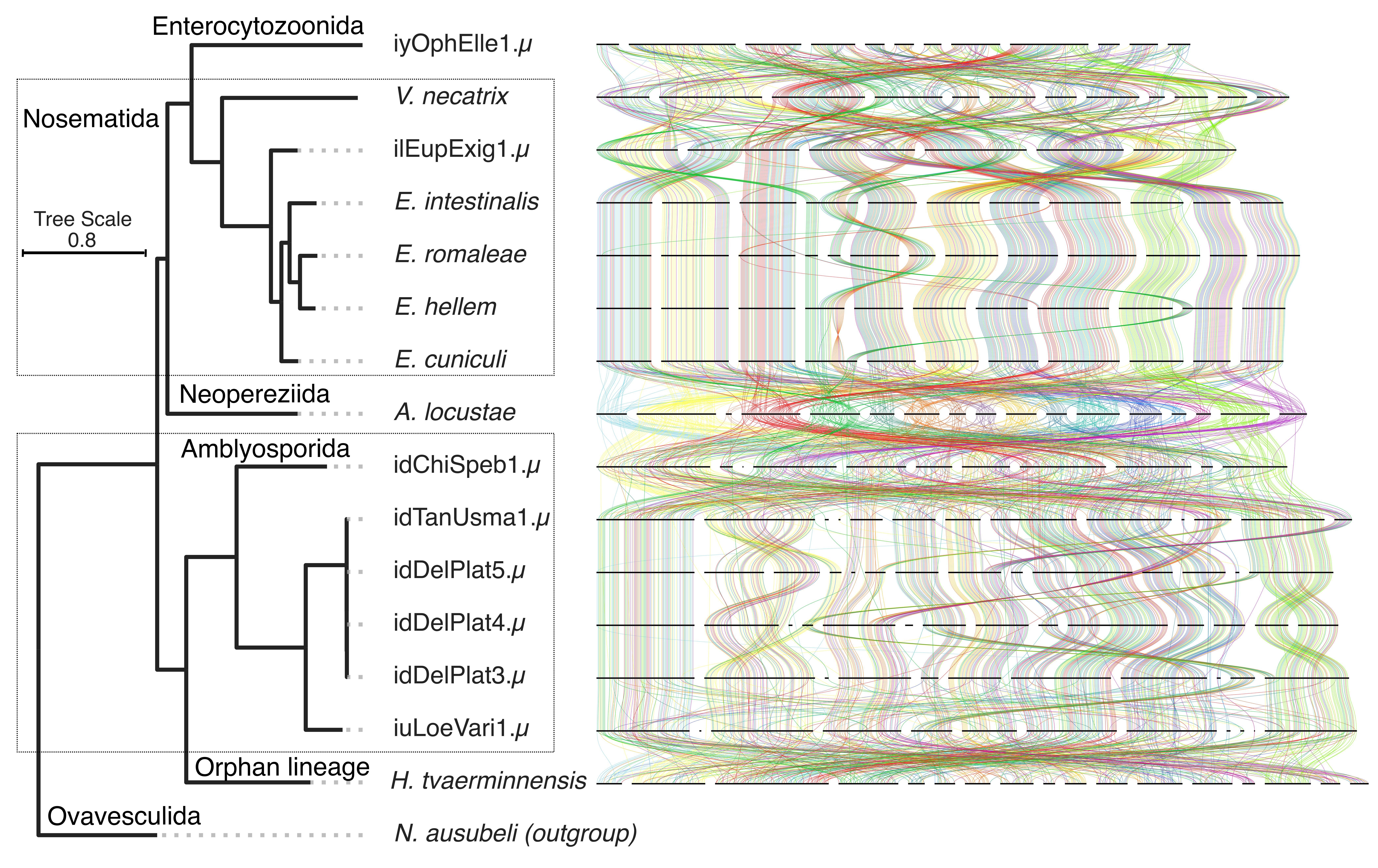

Supplement: S11 Fig — Genome-wide synteny plots of all available chromosomal microsporidian genome assemblies. Each line represents a single-copy BUSCO (microsporidia_odb10) [76]. BUSCOs are painted by their chromosomal position in A. locustae. The data underlying this figure can be found in File Collection 5 at https://doi.org/10.5281/zenodo.17251512. Figure was generated by using ribbon plot scripts from https://github.com/conchoecia/odp [109] and ToyTree [73], and manually annotated using InkScape (version 1.2.2). (PNG) [file pbio.3003446.s021.png]

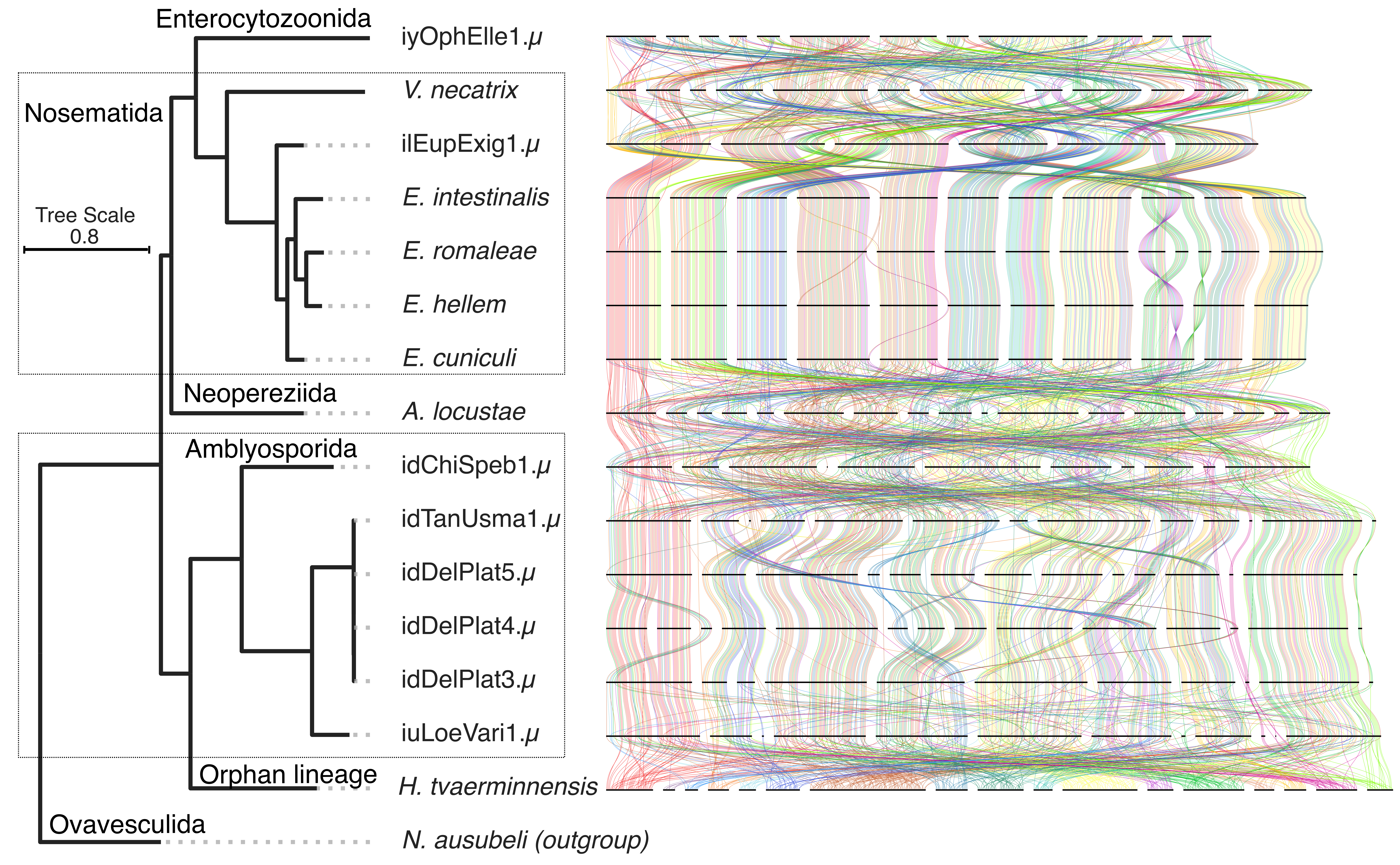

Supplement: S12 Fig — Genome-wide synteny plots of all available chromosomal microsporidian genome assemblies. Each line represents a single-copy BUSCO (microsporidia_odb10) [76]. BUSCOs are painted by their chromosomal position in H. tvaerminnensis. Figure was generated by using ribbon plot scripts from https://github.com/conchoecia/odp [109] and ToyTree [73], and manually annotated using InkScape (version 1.2.2). (PNG) [file pbio.3003446.s022.png]
