# Supplementary material for: Forty new genomes shed light on sexual reproduction and the origin of tetraploidy in Microsporidia
Source: PLoS Biol. 2025 Oct 21;23(10):e3003446. doi: 10.1371/journal.pbio.3003446 (PMC12558613; doi:10.1371/journal.pbio.3003446)
Supplement: Attachment — Submitted filename: Response to Reviewers and Addressing Policy Concerns.pdf [file pbio.3003446.s026.pdf]

We would like to thank the editors and the reviewers for their time and comments. We feel this has greatly improved our manuscript.

Reviewer #1:

No comments.

We thank the reviewer for their time and consideration.

Reviewer #2:

Although the authors do address some of my concerns there are some that they don't. My biggest remaining concern is the lack of protein predictions and annotations. Although the authors don't use genome wide protein predictions them in their analysis, a large value of a paper that assembles novel genomes, is that the genomes are used as a resource, and the prediction and annotation of proteins is a large part of that. I also don't understand the data on the protein prediction numbers the authors provide. It seems like AUGUSTUS for many of the genomes is predicting 0 proteins. Presumably for their BUSCO analysis they are relying on AUGUSTUS predictions, so its unclear to me why their predictions are so low. My other remaining concerns are listed below.

We apologise for not addressing this concern previously, and thank the reviewer for pointing this out. We have now included the GeneMark-ES .gff annotation files generated as part of the BRAKER2 pipeline in our Supporting Information, as they are most in line with the RNA-seq based annotations on Genbank. Please see Supporting Information Section 1, File Collection S14.

1. The authors claim that the genomes are clonal infections but they still don't provide convincing data to support that. Their marker scan approach is relying on 1000 bp long sequences (this is from the cited paper, the authors should include this information in their methods) and if only 10 percent of the genome might be sequenced, this might result in no ribosomal sequence or only a fragmented ribosomal sequence. Their genome scan results also likely wouldn't be able to detect if only 10 percent of the genome was from a different species. Their blob tools analysis could potentially deal with a partial cooccurring genome. The authors state: " A mixed infection of multiple microsporidians would display a distinct pattern in the BlobToolKit plots - especially given that the two (or more) microsporidians would likely differ in read depth (because of different levels of infection)," Here are a couple of examples of non-chromosomal genomes and the range of coverages that they see. Do the authors have evidence that a co-infection that is at 10% of the main infection would not have any contigs included within the low range of coverage?

iyAmbProt1.µ 2.219 - 41.448

ihCicViri2.µ 21.3 - 345.6

Although these genomes may indeed be from single clonal infections, I do not believe the authors have provided evidence to support their new claim "we determined that all of our forty microsporidian genomes are derived from single, clonal infections." I do think this is a hard problem and the authors do not necessarily have to solve it, but If the authors are not going to convincingly address this, adding a sentence to the discussion or methods about the limitation of analysing microsporidia genomes from metagenomic data would be helpful.

We thank the reviewer for pointing this out, and agree with them that our initial response did not address the problem sufficiently. We note that for genomes where read depth was sufficient to estimate ploidy, gene coalescence analyses (such as Fig 3. and Fig. 4 for tetraploid genomes, and Fig. 7 for diploids) would have identified if an infection consisted of two different species of Microsporidia. In the same genomes with high coverage, GenomeScope2 analyses would have highlighted the presence of multiple strains of the same species as well, if that were the case. This is because two co-infecting strains are unlikely to be maintained at identical loads. However, we agree with the reviewer that this would still not address multi-strain infections in genomes with low coverage/where ploidy estimation was not possible. So, we have now amended the text to state this clearly as a limitation of our study. Please see lines 782-799 in the document with changes tracked.

2. The authors are still rooting their tree based on Metchnikovella, without an explanation why. This doesn't effect their results, but displaying the tree this way will be confusing as Mitosporidium is considered the outgroup to metchnikovella and the microsporidia.

We apologise about this oversight. We have now amended our tree to be properly rooted at *Mitosporidium daphniae*. Please see the amended Fig. 2.

Reviewer #3:

I have evaluated the revision, and I think the authors have done a decent job of addressing the previous comments. I note that the responses are somewhat hard to follow because the line numbers do not jive with the rebuttal.

We thank the reviewer for their comments, and we apologise about the line number mismatch.

I think the authors should keep in mind that actually it is very easy to have a situation where you have a completely homozygous diploid. There are many eukaryotes where haploid selfing occurs and generates completely homozygous diploids. They do acknowledge that a completely homozygous diploid is impossible to differentiate from a completely homozygous tetraploid or haploid. I also stick behind my statement about ANI, but we can disagree on this opinion and this doesn't influence the results in this paper.

We thank the reviewer for pointing this out. We have now amended the text to include the caveat of haploid-selfing in eukaryotes. Please see line 741 in the document with tracked changes.

Congratulations to the authors.

We would like to thank the reviewer for their time and consideration.

The Editor

a) Please address the remaining points from the reviewers…

We hope our comments above have sufficiently addressed the reviewers' concerns.

b) ...of which the Academic Editor said "I think that reviewer #2 has a valid point that it can be hard to discern if these are clonal vs polyclonal infections. I don't think it takes away from their major conclusions to add this as a caveat given that extent of analysis. As for the protein predictions, I think you can ask that they also address this and ensure that the methods they have used are sufficiently documented that this can be repeated by others working in the field." I've included that in case it's helpful.

We agree with the Academic Editor and the reviewer, please see response to Reviewer 2. We have now amended the text to state this clearly as a limitation of our study. Please see lines 782-799. We have also included the protein predictions as requested. Please see Supporting Information Section 1, File Collection S14.

c) Please address my Data Policy requests below; specifically, we need you to supply the numerical values underlying Figs 1, 2ABCDE, 3AB, 4, 5, 6A, 7, 8ABC, 9AB, and all of the Supp Figs, either as a supplementary data file or as a permanent DOI'd deposition. I note that you already have an associated Zenodo deposition (https://urldefense.proofpoint.com/v2/url?u=https-3A__doi.org_10.5281_zenodo.15364388&d=DwIBaQ&c=D7ByGjS34AllFgecYw0iC6Zq7qIm8ucIZFI0SqQnqBo&r=D1_49WsfVsssHYZBWWGnrfnH7uUQpSsDXLcZ3aTE1YY&m=nl3lLeqBqEyvP7WecjmwXm90ppQEA14LoxgSoDS_RgxEPMA-sw3rXBsrc0p53va1&s=6PL-NBO9Andfv30_hhnqSivnWHW_ZLI023Xf-aFa3KE&e= <https://urldefense.proofpoint.com/v2/url?u=https-3A__doi.org_10.5281_zenodo.15364388&d=DwIBaQ&c=D7ByGjS34AllFgecYw0iC6Zq7qIm8ucIZFI0SqQnqBo&r=D1_49WsfVsssHYZBWWGnrfnH7uUQpSsDXLcZ3aTE1YY&m=nl3lLeqBqEyvP7WecjmwXm90ppQEA14LoxgSoDS_RgxEPMA-sw3rXBsrc0p53va1&s=6PL-NBO9Andfv30_hhnqSivnWHW_ZLI023Xf-aFa3KE&e=>). Please could you confirm whether the data and code in this deposition are sufficient to recreate the Figures (main and supplementary)?

We have amended our Supporting Information to ensure all the data underlying all the figures (main and supplementary) is directly available in the Supporting Information, or can be easily regenerated using supplied detailed instructions. Please see the following table for the main figures in particular. Under each figure, we have now directly pointed the reader to where the underlying data is found, and how the figures can be easily recreated.

Figure	Data underlying figure supplied (Supporting Information Section, specific item)	Any associated custom scripts that generated plotted data
Fig. 1	- Host metadata table (Supporting Information Section 1, Table S1).	Not applicable. The tree and associated bar graph were plotted using ToyTree, and the whole figure was annotated manually using Inkscape. These tools are now cited appropriately in the figure legend.
Fig. 2	- Genome assembly statistics table (Supporting Information Section 1, Table S1).<br>- Newick string of phylogeny (Supporting Information Section 2, Text S1).	Not applicable. The data shown in the bar graphs were derived by running assembly-statistics, BUSCO, RepeatModeler and RepeatMasker. The tree and associated bar graphs were plotted using ToyTree, and the whole figure was annotated manually using Inkscape. These tools are now cited appropriately in the figure legend.
Fig. 3	- Haplotype phylogenies from which the branch lengths plotted in A were derived. (Supporting Information Section 3, File Collection S15).<br>- BUSCO annotations used in Oxford Dot Plot in B (Supporting Information Section 1, File Collection 5).	Python script used to extract pairwise branch lengths from each haplotype phylogeny and plot histograms seen in A (Supporting Information Section 3, Script S1).
Fig. 4	- Collated "Approximately Unbiased" phylogenetic test results used to populate heatmap (Supporting Information Section 3, Text S2).	Not applicable. The data shown in the heatmap was derived by running BUSCO, IQ-TREE, and the Approximately Unbiased Test (as described in the relevant section). The tree and associated heatmap were plotted using ToyTree and matplotlib. The whole figure was annotated manually using Inkscape. These tools are now cited appropriately in the figure legend.
Fig. 5	- The genome can be	Not applicable. The data shown in the coverage

	found in Supporting Section 1 File Collection S12, along with its BioSpecimenID (Table S1), which can be used to retrieve the associated PacBio reads on NCBI.	track was derived by mapping the PacBio reads against the genome using minimap2, and extracting read depth data using samtools and bedtools. The data shown in the GC data track can be derived by running seqkit fx2tab on the genome. The data shown in the nucleotide identity track can be regenerated by mapping the homelogous copies of chromosome 1 to one another using minimap2, and extracting the resultant nucleotide identity. The plots were generated using matplotlib. The whole figure was annotated manually using Inkscape. The tools and approaches used are now described and cited appropriately in the figure legend.
Fig. 6	- The genome can be found in Supporting Section 1 File Collection S12, along with its BioSpecimenID (Table S1), which can be used to retrieve the associated Hi-C reads on NCBI.	Not applicable. The data shown in the figure was generated by mapping the Hi-C reads to the genome using the tools cited in the relevant section (pipeline: https://pipelines.tol.sanger.ac.uk/curationpretext/1.5.0). The figure itself was generated using PretextMap and InkScape. The tools and approaches used are now described and cited appropriately in the figure legend.
Fig. 7	- Age distributions generated by WGD (Supporting Information Section 3, File Collection 16).	Not applicable. The data shown in the histograms was derived by running wgd. The histograms were plotted using matplotlib. The figure was annotated manually using Inkscape. These tools are now cited appropriately in the figure legend.
Fig. 8	- BUSCO annotations (Supporting Information Section 1, File Collection S5).	Not applicable, the tool used is cited in the relevant section and the Github repository is linked.
Fig. 9	- BUSCO annotations used in ribbon plot (Supporting Information Section 1, File Collection S5).	Not applicable, the tool used is cited in the relevant section and the Github repository is linked.
Fig. 10	Not applicable	Not applicable

d) Please cite the location of the data clearly in all relevant main and supplementary Figure legends, e.g. "The data underlying this Figure can be found in S1 Data" or "The data underlying this Figure can be found in

https://urldefense.proofpoint.com/v2/url?u=https-3A__zenodo.org_records_15364388&d=DwIBaQ&c=D7ByGjS34AIlFgecYw0iC6Zq7qlm8ucIZFI0SqQnqBo&r=D1_49WsfVsssHYZBWWGnrfnH7uUQpSsDXLcZ3aTE1YY&m=nl3lLeqBqEyvP7WecjmwXm90ppQEA14LoxgSoDS_RgxEPMA-sw3rXBsrc0p53va1&s=r_dO5h3ULniKgNGx89Z6nfVZ3OG7v_lmNAHI-zi1dlc&e=
<https://urldefense.proofpoint.com/v2/url?u=https-3A__zenodo.org_records_15364388&d=DwIBaQ&c=D7ByGjS34AIlFgecYw0iC6Zq7qlm8ucIZFI0SqQnqBo&r=D1_49WsfVsssHYZBWWGnrfnH7uUQpSsDXLcZ3aTE1YY&m=nl3lLeqBqEyvP7WecjmwXm90ppQEA14LoxgSoDS_RgxEPMA-sw3rXBsrc0p53va1&s=r_dO5h3ULniKgNGx89Z6nfVZ3OG7v_lmNAHI-zi1dlc&e=> ”**

Please see above. We have amended our figure legends to point at where the underlying data associated with each figure can be found.

e) I note that (unlike in earlier versions) the supplementary Figs and Tables are currently only accessible as part of very large zipped folders – please submit these as separate files, and include their legends in the manuscript file. It’s fine for the other files (non-Fig, non-Table) to simply be provided in the Zenodo deposition.

We apologise about this. We have now amended our Supporting Information structure so that they can be accessed easily, and not as part of large zipped folders. We have also included figures and tables where possible, as part of our submission on the online portal.

f) Please make any custom code available, either as a supplementary file or as part of your Zenodo deposition.

Please see above. We have now provided all custom code used as part of our Zenodo deposition.

g) Please include the URLs of your funders in the Financial Disclosure statement.

We have now provided the requested URLs as part of the Financial Disclosure statement.
