# Supplementary material for: Forty new genomes shed light on sexual reproduction and the origin of tetraploidy in Microsporidia
Source: PLoS Biol. 2025 Oct 21;23(10):e3003446. doi: 10.1371/journal.pbio.3003446 (PMC12558613; doi:10.1371/journal.pbio.3003446)
Supplement: Attachment — Submitted filename: Response_to_Reviewers.docx [file pbio.3003446.s025.docx]

**Forty New Genomes Shed Light on Sexual Reproduction and the Origin of Tetraploidy in Microsporidia**

Amjad Khalaf*^1^, Chenxi Zhou^1,2^, Claudia C Weber^1^, Emmelien Vancaester^1^, Ying Sims^1^, Alex Makunin^1^, Thomas C Mathers^1^, Dominic E Absolon^1^, Jonathan MD Wood^1^, Shane A McCarthy^1^, Kamil S Jaron^1^, Mark Blaxter^1^, and Mara KN Lawniczak^1^.

1 Tree of Life, Wellcome Sanger Institute, Wellcome Genome Campus, Cambridge CB10 1SA

2 Department of Genetics, University of Cambridge, Cambridge CB2 3EH, UK

* Corresponding author E-mail: [ak37@sanger.ac.uk](mailto:ak37@sanger.ac.uk)

**We would like to thank the reviewers and the editor for their thoughtful comments, suggestions, and their input. We feel that this has greatly helped us sharpen the manuscript and shape its narrative.**

## Reviewer #1:

**The paper by Khalaf and colleagues provides a comprehensive analysis of 40 microsporidian genomes derived from long-read sequencing datasets of infected animal hosts. The study is robust, with results that are generally well-supported by the provided data and analyses. Below, I offer suggestions to improve the paper's structure, readability, and scientific rigor, along with specific technical comments.**

**Note that the absence of line numbers and the placement of figure legends (separated from figures and embedded mid-text) made the review process unnecessarily challenging, and I recommend addressing this for clarity.**

We thank the reviewer for their kind comments, their suggestions, and their time. We also apologise for the absence of line numbers, we have now amended this in our resubmission. As for the placement of the figures and figure legends, we have followed PLOS Biology’s submission guidelines, which state:

- *Figure files: Upload separately. Do not put figure files in the manuscript body.*
- *Figure captions: Insert immediately in the manuscript text after the first paragraph in which the figure is cited.*

**General Comments:**

***	Streamline Content for Conciseness and Broader Appeal: While the dataset is extensive, the paper could be significantly shortened by consolidating analyses addressing similar questions. For instance, the phylogenetic and species definition analyses could be combined into a single, concise paragraph, as they are closely related. Similarly, analyses of inter-species versus intra-haplotype rearrangement/recombination both explore genomic diversity in microsporidia and should be merged into one section. Reducing overly detailed discussions of niche interest (e.g., specific phylogenetic nuances) would enhance accessibility for the broader readership of PLoS Biology.**

We thank the reviewer for their suggestions, and apologise about the lack of streamlining and the length of the manuscript. We have now significantly shortened three sections of the manuscript, and removed some of the more niche points. Details of enacted changes are as follows:

1. We have moved the discussion about host sex, and any sex distortion possibilities of microsporidians to the Supporting Information. See lines 143-145 in the revised document. As part of this, we have also moved panel B of Figure 1 to the Supporting Information.
2. We did not combine section 1 “Forty new microsporidian genome assemblies” and section 2 “Operational taxonomic unit classification of species suggests autotetraploidy in Microsporidia”, as we felt that they are distinct topics despite both relying on phylogenetics. However, we have significantly shortened section 2, moving many of the details on methodology to the Supporting Information.
3. We completely moved section 7 “Segmental duplications are common in Microsporidia” to the Supporting Information, along with Figure 9. We replaced it with a concise paragraph explaining how ploidy was called, and highlighting the existence of these segmental duplications in section 1 “Forty new microsporidian genome assemblies”. See lines 188-197 in the revised document.
4. We merged section 8 “Evidence for between-homeologue rearrangement in some microsporidian genomes” and section 9 “Large-scale chromosomal rearrangements between microsporidian groups” into one section titled “Evidence for rearrangements between homeologues and between genomes in Microsporidia”. As part of this, we significantly moved parts of the former section 9 “Large-scale chromosomal rearrangements between microsporidian groups” to the Supporting Information.

We hope these large-scale changes help make the manuscript more streamlined and accessible to a broader audience.

***	Clarify "Compartments" Terminology: The use of "compartments" in the context of eukaryotic genomes typically refers to A/B compartments—genomic regions with distinct gene/repeat enrichments and conformational differences tied to the cell cycle. The Hi-C data presented suggests compartmentalization, but it's unclear whether these reflect standard A/B compartments, as seen in other eukaryotes, including symbiotic or pathogenic fungi. The authors should investigate and explicitly compare their findings to known A/B compartmentalization to avoid confusion.**

We agree that the use of the word “compartment” could be confusing. The compartments referred to in the manuscript are likely two nuclei, not the same as the classical chromatin A and B compartments which occur within one nucleus. To increase clarity, we have now replaced the word “compartment” with “unit” throughout the whole manuscript. For instance, we have now amended the quoted text to say “We show that one tetraploid genome is organised into two **diploid** **units**, likely the nuclei of the diplokaryon” - highlighting that this is a partition of a whole genome into two physical units (each of which contains two subgenomes). We have also enacted this change in the Hi-C figure, which previously contained the word “compartment”.

***	Acknowledge Prior Hypotheses on Microsporidian Sex and Ploidy: The paper's major claims, such as the proposed sexual reproduction model and diploid/tetraploid cycles, build on earlier hypotheses that are not cited. For example, Pelin et al. (2016) and Corradi (2015) discuss diploid-tetraploid cycles and potential karyogamy in microsporidia, suggesting mechanisms for genetic diversity. These seminal works must be referenced to contextualize the current findings. The authors should clarify whether their data supports, refines, or challenges these hypotheses. Additionally, claims of sexual reproduction should be substantiated with population genetics analyses, which are absent here but have been explored elsewhere (e.g., in studies not cited in this manuscript).**

We apologise about this oversight, and thank the reviewer for pointing this out. We have now made the following changes in our citations and claims:

1. We have cited Pelin et al. 2015 to particularly say that they proposed that tetraploids are organised as diplokarya, and that our data supports their hypothesis. See Introduction lines 78-83 and Discussion lines 563-565 in the revised document.
2. We have cited Corradi (2015) to specifically say they proposed a model of haploid-diploid or diploid-tetraploid cycling in microsporidian reproduction, followed by a period of clonal propagation - and that our data supports their hypotheses. See Introduction lines 78-83 and Discussion lines 563-565, and Discussion lines 580-582.
3. We have cited all of: Angst et al. 2022, Sagastume et al. 2016, Krebes et al. 2014, Cuomo et al. 2012, Haag et al. 2013, Ironside 2013, and Sagastume et al. 2011; to say that they have carried out population-level genetic sampling, and that their evidence also supports the occurrence of sexual reproduction in Microsporidia. See Introduction lines 71-72.

We hope these changes provide better treatment of the literature, and emphasise that our manuscript is building on previously suggested hypotheses and pieces of work.

**Specific Comments:**

***	Page 7, Compartmentalization: The statement, "We show that one tetraploid genome is organised into two compartments, likely the nuclei of the diplokaryon," requires clarification. Do these Hi-C-defined compartments show differential enrichment in transposable elements (TEs), BUSCO genes, or other features, as expected in A/B compartments? The authors should compare their findings to known eukaryotic compartmentalization to ensure accurate terminology.**

We have addressed this concern under the responses to the general comments.

As for differences between the two diploid units, we have highlighted in Figure 5 and 6 in the revised document that they are nearly identical. No large differences in genome architecture and content are observed. Under the proposed model that the two units represent a partitioning of the tetraploid genome into two recombining diploid nuclei, this is to be expected.

***	Page 9, Genome Data Accessibility: The paper lacks a clear summary of the 40 genomes' characteristics. I recommend including a table detailing genome statistics (e.g., size, scaffold number, N50), sequencing methods (e.g., Hi-C, telomere resolution), and quality metrics. Additionally, explain why Hi-C was applied to some genomes but not others and outline the sequencing-to-scaffolding pipeline to provide context for methodological choices.**

All the requested information is already provided in Supporting Information Section 1, specifically Supporting Information Table S1. See Results Section 1, line 150 where such information is referenced. Genome spans and N50s are also reported in Figure 2. We have opted to leave the full table in the Supporting Information as it would be too large of a table to include in the main text. We have now amended the text to explicitly say “The full list of hosts, their recovered microsporidian genome assemblies, **and associated genome summary statistics** are given in Supporting Information Section 1.” See Results, section 1, line 145.

We also have explained why Hi-C was applied to some genomes and not others in the Materials and Methods section, specifically the Data subsection. See lines 625 to 633. In brief, the typical Darwin Tree of Life process relies on using different parts of one individual, or two closely related individuals, to generate long-read DNA sequencing data and Hi-C short-read sequencing data. Because of this, specimens identified as microsporidian-infected from long-read sequencing data did not often have Hi-C data available. So, we went on to do further sampling of flying insects using Malaise traps to ensure we were able to generate Hi-C data for a subset of the microsporidian genomes we explored.

***	Page 11, Figure and Legend Placement: Embedding figure legends mid-text, with figures placed at the end, hinders readability. Figures and their legends should be integrated within the main text at relevant points to facilitate review and comprehension.**

We apologise about the inconvenience. We have followed PLOS Biology’s submission guidelines, which state:

- *Figure files: Upload separately. Do not put figure files in the manuscript body.*
- *Figure captions: Insert immediately in the manuscript text after the first paragraph in which the figure is cited.*

***	Page 12, Annotation Pipeline: The bacterial annotation pipeline is surprising given microsporidia's eukaryotic nature. Although introns are rare and small, a eukaryotic annotation pipeline is standard and should be employed. Justify the use of a bacterial pipeline or revise the methodology.**

We thank the reviewer for pointing this out. We had previously stated the limitations of Prokka in our methods section, and we had explained that we elected to use it given: a) Microsporidia’s rare and few introns, and b) the fact that no functional annotations were required in this piece of work.

However, we have now also explored other annotation options which are more appropriate for eukaryotes (namely Augustus and Braker2). It is clear that there is a wide discrepancy between different tools (even eukaryote-appropriate ones), as illustrated in the figure below where each position on the x-axis indicates a different microsporidian genome. While Prokka has the largest variance in number of genes called overall, there are apparent categorical differences between (e.g.) Augustus and BRAKER.

Given this discrepancy, we have made the choice of removing the annotations completely from our manuscript. Previously, Prokka was used to:

1. Estimate coding sequence counts roughly. These results are shown in Figure 2, and are used in a rudimentary correlation with Pagel’s tree transformations [topology (λ), branch-lengths (κ) and root-tip distance (δ)] only. We have now removed these completely from the figure and the correlation section. See lines 768 - 773 where we explain why Prokka was removed from the manuscript.
2. The section showing a lack of rediploidisation evidence (now Figure 7). **We have now amended this analysis to rely on BUSCOs only**, where predictions used the embedded Augustus predictor**.** See the updated Figure 7, showing similar results to the previous version.

All our other analyses used BUSCOs, and we have now explicitly stated that in the manuscript.

***	Page 12, Transposable Elements and rRNA: The paper notes correlations between retroelement, DNA transposon, and helitron loads with genome size but lacks detail on their genomic distribution. Are TEs or rRNA operons enriched in specific chromosomal regions, such as subtelomeres, as observed in Encephalitozoon? This should be explored and discussed.**

We thank the reviewer for suggesting this interesting avenue of exploration. After examination, we find that Encephalitozoons are the exception in Microsporidia. In other chromosome-level genomes, including publicly available ones not generated by our study, the majority of chromosomes display different patterns. This can be the total opposite pattern to that of Encephalitozoons with repeats and rRNAs clustering in the centre of chromosomes. There are also instances where it is difficult to discern any pattern. Please see lines 206-210 in the revised document where we mention this in the main text. We have also added all our TE and rRNA landscape figures to the Supporting Information Section 2.

***	Page 13, Species Definitions: Defining species based on branch length is problematic, as it risks oversimplification (e.g., analogous to grouping marsupials and mammals as conspecific). Species delimitation in microsporidia should incorporate population genetics approaches to assess genetic exchange, as asexual lineages may not align with traditional species concepts. The authors should acknowledge these limitations and consider integrating population genetics data to support taxonomic claims.**

We agree with the reviewer that more population data would be helpful in species delineation. The approach we use in the manuscript by default relies on multiple genomes for the same species when they are present, and these were included in computing our species delineation thresholds. For instance, the following species have three or more publicly available genomes, and so this small set of population-level data was used in assessing how “similar” individuals belonging to the same species are: *Enterocytozoon hepatopenaei*, *Vairimorpha ceranae*, *Ordospora colligata*, *Encephalitozoon hellem*, *Encephalitozoon cuniculi*, *Anncaliia algerae*, *Hamiltosporidium tvaerminnensis*, *Spraguea lophii*, *Nematocida parisii*, and *Nematocida ausubeli*.

We disagree that the application of our approach would declare marsupials and eutherians as belonging to the same species-like group. Our method relies on using branch length as a proxy for similarity between individuals known to belong to the same species, and applying that benchmark to individuals with no morphological data - allowing us to say how similar they are to other known species. In this approach, marsupials and eutherians would be clearly declared as not belonging to the same species, given that the branch length distance between any two individual eutherians is smaller than the branch length distance between a eutherian and a marsupial.

We agree however that the traditional species concept may not apply to all microsporidian species, especially if some of them are purely asexual. So, we have now amended the text to reflect this uncertainty, and that we use them solely as a proxy for roughly delineating species. See lines 228-231 in the revised document.

***	Page 15, Nomenclature: Designations like "idChiSpeb1.μ" are opaque and confusing. If a species name exists (e.g., for the sample from Chironomus sp.), it should be used consistently to improve clarity.**

While we agree with the reviewer that our designations are not as clear as listing the host species name directly, ToLIDs are the names the host genome assemblies are released under and the names associated with their BioSample IDs. For example, see <https://www.ncbi.nlm.nih.gov/datasets/genome/?taxon=111903>.

ToLIDs are a valid designation linked to a variety of metadata on NCBI. They have also been previously used in multiple publications (see <https://www.pnas.org/doi/abs/10.1073/pnas.2115639118> for example). The “.µ” suffix is only used to denote microsporidia in the manuscript, as clarified in the methods section. The full name each assembly will be released under is outlined in the Supporting Information, with such a formula being the blueprint “HostToLID.MicrosporidiaGenus_MicrosporidiaSpecies”.

## Reviewer #2:

**Microsporidia are a large group of intracellular parasites that infect many species of animals. Their genomes have been of interest as they have the smallest known eukaryotic genomes, but their genomic structure has not been thoroughly investigated due to only have a few species with chromosomal level assemblies. Here the authors take advantage of genome reads from serendipitously infected insects to assemble 40 genomes, representing 17 species. The authors use these genome assemblies to investigate ploidy, genome structure, and provide evidence for sexual reproduction. Overall, this is an interesting paper and the high quality microsporidia genome assemblies will be a useful resource.**

We thank the reviewer for their kind comments, their suggestions, and their time.

**Major points:**

**1. The authors' claim "They allowed us to revise microsporidian phylogeny, notably changing the position of Glugeida to the sister group of the ancestor of the 'orphan lineage and Amblyosporida" is unsupported by the provided data. There needs to be support values provided for the nodes of the tree in figure 2 and ideally more than one phylogenetic approach would be used. The authors use terms like "clustering robustly" and "We confidently placed" , but it is unclear what these descriptions are supported by. The authors choice to use metchnikoviids as the root is not explained, as most groups show that Mitosporidium daphnae is sister to the metchnikoviids and microsporidia. The nodes are also very difficult to see as they are compressed. I would recommend creating a separate version of this tree for the main figure that is just species and not strains. The full tree can then be a supplemental figure.**

We apologise about the lack of support in our Figure 2, and thank the reviewer for pointing this out. We have now amended Figure 2 to include support values. We have also toned down one of our claims, namely the one about Gluegeida’s position, to reflect that it is proposed by a node with less than 95% support (89% support instead). See lines 214-218 in the revised document. The remainder of our claims are all supported by nodes with 100% support.

As for the outgroup, our phylogeny includes both *Mitosporidium daphniae* and *Metchnikovella incurvata.* The two confidently occupy a basal position in our phylogeny, in agreement with other published phylogenies. *Mitosporidium daphniae* is often considered a microsporidian, given that it possesses an infective apparatus similar to that of other microsporidians, and is typically referred to as a “short-branch” microsporidian. Please see Bojko et al. (2022) “Microsporidia: a new taxonomic, evolutionary, and ecological synthesis” and Bass et al. (2018) “Clarifying the Relationships between Microsporidia and Cryptomycota”. Furthermore, other published phylogenies include both *Mitosporidium* and *Metchnikovella*, using the latter as their outgroup. See Bojko et al. (2022). Thus, we elected to do the same.

We apologise about the resolution of the image. We have now generated a smaller phylogeny, excluding strain information as suggested, to be in the main text. See the revised Fig. 2. The phylogeny with all the genomes available has been moved to the Supporting Information. We have also enhanced the main text figure further to ensure all the nodes are clearly visible. The newick string itself is now also available in the Supporting Information, in order for readers to be able to investigate specific nodes in detail with ease.

2. **The number of genes predicted which are shown in figure 2 seem unreasonably high. Some species of microsporidia are reported to have less than 2000 protein coding genes, so the reporting of at least 10 species having over 20,000 proteins is unexpected. What is the explanation for this? Is this different variants for the same gene being predicted multiple times? Or is this just very short proteins? Other methods besides Prokka that have previously been used on microsporidia genomes should be investigated (see the following paper for examples: DOI: 10.1111/jeu.13038.) The authors state that "no functional annotations were required in this piece of work", but gene prediction affects analysis such as figure 4a and figure 8 which some species have 100 gene pairs vs 1000 gene pairs. Also for these genomes to be useful resource, the annotations along with the genome assemblies should be deposited in a central repository such as NCBI.**

We thank the reviewer for pointing this out. We have addressed this alongside Reviewer 1’s concerns with Prokka. In brief, we have now tested other annotation approaches (Augustus with a microsporidian model, and Braker2). We found that as discussed previously in the literature, these different methods show large discrepancies between them, and between the GenBank annotations that rely on RNA-seq. Given that the coding sequence annotations were used to make a very small point in our manuscript, we have now made the decision to exclude protein sequence annotations completely.

All analyses, including Figure 4A prior to revisions (now Figure 3A), rely on BUSCOs. We have also now explicitly stated that BUSCOs were used in all analyses. The only previous exception was the rediploidisation section, which used the Prokka annotations. We have now amended this to use BUSCOs as well, and it yields the same results as in the previous version of the manuscript.

**3. There are very high levels of duplicated buscos (up to close to 100%) in some assemblies. Is this just because some of the assemblies are haploid resolved? This needs to be explained.**

We apologise about the confusion. As pointed out by the reviewer, this is because some of our genome assemblies are not purged, and thus retain haplotypic duplication. As such, BUSCO duplication may be up 100% if all two or four haplotypes are retained. This was previously addressed in the methods sections. We have now amended the main text to state this early on. Please see lines 165-169 in the revised document. The text now explicitly states “The eight largest genome assemblies are unpurged due to their read depth being too low to run purge_dups, or because of the presence of severe between-haplotype rearrangements, or due to the lack of Hi-C data; and thus retain haplotypic duplication (which also results in the high BUSCO duplication scores observed in Fig. 2).”

**4. How the paper deals with purged genomes is confusing. A better explanation of how and why genomes were purged would be helpful to add.**

We apologise about the confusion. This is addressed alongside the previous concern, see the amended text.

**5. The authors do not appear to take any steps to confirm that the microsporidia reads from their samples come from a single species. Co-occurrence of different microsporidia species in mosquitos has been shown to be ~10% (https://urldefense.proofpoint.com/v2/url?u=https-3A__doi.org_10.1111_1755-2D0998.13205&d=DwIBaQ&c=D7ByGjS34AllFgecYw0iC6Zq7qlm8uclZFI0SqQnqBo&r=D1_49WsfVsssHYZBWWGnrfnH7uUQpSsDXLcZ3aTE1YY&m=A57Qr1zvwmHyxPaS5ubqO6xFXXAqR2M8IXfbr_CBPPT0bxY6LKgIudCnWwKQakWw&s=uB29zYycGhJ_6aAQqPBvqNwkXVC23V-vGqNmy_ydd_s&e= ). For the non-chromosomal level genomes, some criteria needs to be used to determine whether the microsporidia reads in each sample are from the same species.**

We apologise for the lack of clarity about this. Effectively, all our microsporidian genomes come from single, clonal infections. There are multiple checkpoints where this is confirmed:

1) The initial screen for microsporidian reads relies on fishing out ribosomal sequences from arthropod reads. Had there been multiple species of microsporidia, this would have emerged in this initial screen. Please see <https://wellcomeopenresearch.org/articles/9-33>.

2) A mixed infection of multiple microsporidians would display a distinct pattern in the BlobToolKit plots - especially given that the two (or more) microsporidians would likely differ in read depth (because of different levels of infection), and in sequence composition.

3) The GenomeScope2 kmer histograms are consistent with every genome belonging to a singular clonal infection. We addressed this previously in <https://journals.asm.org/doi/10.1128/spectrum.03669-23>. Had there been multiple genomes in one sample, the coverages and the ratios of k-mers would not be as clean as they appear in the Supporting Information.

We have now clarified this in the text by adding a small section to the methods. See lines 711-723 in the revised document.

**6**. **What criteria is used to classify ploidy in an assembly needs to be defined. For example, why is the ploidy of iyOphElle1.µ, not determined, as this is a chromosome assembly?**

Ploidy was estimated in the same way it was done in Khalaf (2024), as per the GenomeScope2 and Smudgeplot guidelines. We have now added a concise paragraph explaining how ploidy was called in section 1 “Forty new microsporidian genome assemblies”. See lines 188-197 in the document.

As for iyOphElle1.µ, whilst this is a chromosomal assembly its read coverage was too low to estimate ploidy reliably as per the GenomeScope2 and Smudgeplot guidelines.

**7. The number of genome assemblies that were screened using blobtools for microsporidia reads is not mentioned and should be.**

We apologise for this oversight. Darwin Tree of Life genome assemblies were screened on a rolling basis, using MarkerScan. To identify our forty genomes, we have examined 1,200 MarkerScan results in total. This has now been added to the methods section, see lines 656-659 in the revised document.

**Minor points:**

**1. I would recommend against using µ as to name the novel microsporidia species genomes. There will certainly be instances where others will use these genomes in applications where Greek letters are not able to be used, and this could lead to confusion. This is a known issue in biology nomenclature and see the following paper for this recommendation in the context of gene names: DOI: 10.1006/geno.2002.6748 . I would suggest using some other abbreviation such as "m", "micro", or "ms" that makes the same point using the Latin alphabet.**

The “.µ” suffix is only used to denote the microsporidian genome assemblies in the **manuscript**. This is clarified in the methods section. The full name each assembly will be released under is outlined in the Supporting Information, with such a formula being the blueprint “HostToLID.MicrosporidiaGenus_MicrosporidiaSpecies”.

**2. Methods are lacking for the primers and genes that were used to identify individuals as microsporidia positive.**

We apologise about this. In the “Data” part of the methods section we now report “The other half was kept at -80°C for later Hi-C sequencing. We identified infected insects by PCR amplification testing of the long read DNA extracts using a microsporidia-specific amplicon panel targeting the small subunit (SSU) rRNA hypervariable V1-V3 regions [115,116]. The primers used were V1F and R30R, with standard protocol as outlined in the literature [115,116]”.

**3. In figure 1A "number of microsporidia genomes" would be more appropriate as the X-axis label and moved underneath the X-axis.**

We have now amended this, please see the updated Figure 1.

**4. Several acronyms and abbreviations are used that are not defined such as MFP, Asm., and OTU.**

We apologise about this. We have now ensured that all abbreviations and acronyms are explained.

**5.** **The paper often uses a lot of jargon that makes the paper less accessible, such as "Operational taxonomic unit classification of species suggests autotetraploidy in Microsporidia". It would be helpful to revise the paper with an eye towards clarity and simplicity that would help non-specialists be able to understand.**

We apologise about this. Operational Taxonomic Units (OTUs) represent groups of organisms which share similarity, similar to a “species”, but not necessarily in line with the Linnaean system. We have elected to use OTUs instead of “species” as morphological and histopathological data will likely be necessary to truly declare two organisms as belonging to the same species. We have now gone through the manuscript and have removed as much jargon as possible.

**6. The scale in figure 3 is too compressed see the branch lengths of .15 that the authors point as delineating species. It would be helpful to use a different scale that allows this level of differences to be visualized.**

We apologise about this. In light of other reviewer requests, we have now removed this figure in its entirety. The phylogeny used to derive the branch lengths is attached in the Supporting Information.

**7. What is being a considered an OTU in this paper not explained.**

Please refer to our response to minor point 5.

**8. The text in figure 4A is too small to read and needs to be increased to at least 8 pt.**

We apologise about this. We have now increased the size of the text labels in what is now Figure 3A.

**9. In figure 5 the clade names at the bottom are upside down and should be flipped so they can be more easily read.**

We apologise about this. We have now fixed the orientation of the labels in what is now Figure 4.

**10. A scale needs to be shown for figure 7.**

While we cannot directly provide a scale, as the colours in the PretextMap are based on a relative scale, we have now added an explanation in the legend for what is now Figure 6. We hope this will be sufficient.

## Reviewer #3:

**Khalaf et al. report on 40 new Microsporidia genomes that were sequenced incidentally as part of the Darwin Tree of Life project. The authors generated rather intact, complete genomes of these parasites and used Hi-C to assess contacts between homologous chromosomes in a genome. The authors use the data to address a large number of questions in Microsporidia biology, such as phylogeny, synteny, and most interestingly the evolution of tetraploidy. The authors show that tetraploids are autopolyploids rather than allopolyploids. A novel hypothesis that the two nuclei inside diplokaryotic cells are actually in some cases tetraploids divided into diploid nuclear compartments is proposed. Overall, this study provides new insights into the biology of these fascinating parasites and is easy to read.**

We thank the reviewer for their kind comments, their suggestions, and their time.

**However, I have a few major criticisms that dampen my enthusiasm. Firstly, this paper really rests on really accurate estimation of ploidy, which is tricky without cytology. For example, it is impossible to distinguish a haploid from a homozygous diploid or tetraploid. The presentation of how ploidy was assigned to these genomes was not sufficient to evaluate how robust the assignment was. Some of this comes up in Figure 9, where there is a mixed signal which the authors ascribe to segmental duplication. To be clear, I don't think the authors did a poor job in their assignments, I just think they need to be clearer about how they were done and whether there was any ambiguity.**

We thank the reviewer for bringing this up, and for critically looking at our results. We have now amended the manuscript to include an explanation of how ploidy was estimated, see lines 188-191 in the revised manuscript. In brief, ploidy was estimated in the same way it was done in Khalaf (2024), as per the GenomeScope2 and Smudgeplot guidelines (excluding genomes with average read depth < 20X) (Ranallo-Benavidez et al. 2020).

As for the high homozygosity individuals, we disagree with the reviewer. It is still possible to identify a diploid as a diploid (and a tetraploid as a tetraploid), even if its heterozygosity is very low. For instance, we were previously able to identify members of the genus *Encephalitozoon* as diploids, despite their heterozygosity being <0.1%, please see Khalaf (2024). We have also now amended the text to state the following: “We note that GenomeScope2 and Smudgeplot rely on heterozygosity for ploidy estimation (Ranallo-Benavidez et al. 2020), and while individuals with low heterozygosity can still have their ploidy correctly estimated, individuals with exactly 0% heterozygosity are likely to be mis-classified.” - see lines 674-677 in the revised document.

**A second issue relates to the Hi-C data. Here, the authors could do a better job in walking us through what is happening. The figure itself is not self explanatory, and while their hypothesis is interesting, it's not clear how well it is supported by the data. How much uncertainty is there in this diplokaryotic model?**

We apologise about the confusion. We have now amended the figure legend to explain, in some detail, how to read the figure. See the revised Hi-C Figure legend, which states the following: “Hi-C contact maps are heatmaps that visualise the frequency of physical contacts between genomic regions in 3D-space. Regions that are closer together physically tend to show more interactions, appearing as darker colors on the map. The strongest signal, in dark red here, is always found along the diagonal, which represents self–self interactions (i.e. each genomic region interacting with itself and nearby regions along the same chromosome). Off-diagonal signals represent interactions between different chromosomes.”

There is no uncertainty that we see the tetraploid genome clustering as two diploid units in the Hi-C data. Our proposal is that those two diploid units are two distinct nuclei. As to why there would be HiC signal between sequences from two different nuclei (e.g.: Chr1A and Chr1C), we propose that this is the result of read mismapping, or compromised membranes between the two proposed nuclei. The latter may occur as a result of the physical fixation process in some of the cells, or as a genuine biological phenomenon. For instance, gaps between nuclei membranes have been noticed in two diplokarya (Sokolova and Fuxa 2008; Nassonova and Smirnov 2005). Similarly, Hi-C signal is observed across nuclei in dikaryotic fungi (Duan et al. 2022). This is now also discussed in lines 568-573.

**Third, the authors run through many different analyses that are interesting, but again require really solid ploidy estimation and phasing/assembly of data into homeologues, such as the bizarre finding of an uneven and rearranged tetraploid. I have few other general comments, but again the paper is pretty cleanly written.**

We thank the reviewer for their kind comments. We hope we have addressed the reviewer’s concern with regards to ploidy estimation above (major point 1).

**1. The authors should probably streamline some of the Results/presentation. For example Figure 1B is not a big result and there is not much they can say about it. Is it worth even mentioning?**

We apologise about the lack of streamlining. We have addressed this alongside Reviewer 1’s concerns. Please see the response to their first general comment.

**2. A new way of defining species is presented using amino acid substitutions per site. This seems more complicated that the recent analysis by Albuquerque and Haag, doi: 10.1111/jeu.12944 using ANI. I'm not sure the field needs a more complicated method that involves more variation in molecular rates.**

We agree with the reviewer that ANI is another valid way to define a species, as shown by Albuquerque and Haag. However, while our approach is more complicated, it relies on pre-established orthology - as opposed to ANI, which assumes that reciprocal blast hits are likely to be orthologous. Additionally, many ANI approaches do not take into account the proportion of the genome that is represented in the reciprocal blast hits which are assumed to be orthologous. Thus, distant genomes which share small fragments with very high identity can appear to be closer than they are according to ANI. This was also our initial approach, but we ultimately departed from it in favour of the current approach, which is more robust and is less susceptible to noise. Furthermore, our current approach makes use of amino acid sequences as opposed to nucleotide sequences, and takes into account evolutionary variation and molecular rates - aiding in its robustness. We have now explained all of this better in the manuscript, see lines 746 - 753.

**3. Figure 4 only uses tetraploids. However, if allopolyploidy occurred between two species that were diploid but homozygous, it could look the same as a diploid.**

We think that finding perfectly homozygous diploid organisms that upon tetraploidy could be mis-classified as diploid instead of tetraploid is an extremely unlikely scenario. Furthermore, a proposed diploid containing two different-species subgenomes would have been detected as an apparent mixed infection by the implicit checks we carried out:

1) The initial screen for microsporidian reads relies on fishing out ribosomal sequences from arthropod reads. Had there been multiple species of microsporidia, this would have emerged in this initial screen. Please see <https://wellcomeopenresearch.org/articles/9-33>.

2) The GenomeScope2 kmer histograms are consistent with every genome belonging to a singular clonal infection. We have addressed this in <https://journals.asm.org/doi/10.1128/spectrum.03669-23>. If a genome is made of two highly homozygous diploids which are highly divergent from one another, the coverages and the ratios of k-mers would not be as clean as they appear in the Supporting Information. See Khalaf (2024) for an example of mixed infections.

**4. Without evidence of deep coalescence between homeologues, is the section "No evidence of recent rediploidisation in Microsporidia" likely to find anything?**

We agree with the reviewer that without evidence of deep coalescence between homeologues, there is no evidence of rediploidisation. However, we note that in the manuscript we do not test for coalescence of homeologues in diploids outside the “No evidence of recent rediploidisation in Microsporidia” section. All our other tests involve tetraploids, and thus this section is necessary for diploids. Furthermore, this section is important as not all homeologues need to be deeply coalescent for there to have been rediploidisation. If the majority of ancient polyploidy signals are lost, coarse approaches which may be able to show ancient polyploidy in a genome (such a phylogeny), may not be sufficient, and we instead may need to look at each paralogous gene pair individually (as we did in that section).

By showing the absence of deep coalescence, across large numbers of BUSCOs, this section highlights that diploid lineages were likely diploid throughout or rediploidised without retaining ancient paralogous signals (by a reductive-division-like process).

We have clarified this in the revised text, see lines 390 - 411.

**5. Figure 12 could use a little polishing as it shows meiosis yielding two products and gametes undergoing mitosis. These simplifications could end up confusing readers.**

We thank the reviewer for pointing this out, and we apologise about the oversimplifications in our figure. We have updated it now to minimise possible confusions.
